# A Manual of Microscopical Technology

**Published:** 1886-03

**Authors:** 


A MANUAL OF MICROSCOPICAL TECHNOLOGY. By Dr. Carl Friedlander. Berlin. Translated by S. J. Howell, M. A., M. D. Buffalo: 12 mo., p., 249. G. P. Putnam's Sons, New York, 1885.

This little volume, although preceded by another translation from the same German original, contains numerous additions in the shape of explanatory foot-notes and is, besides, a good translation. The book is designed as a help to the student and points out in concise form methods and details necessary in properly carrying on microscopic work. But the omission of an index is almost unpardonable. Time is too valuable to be lost in fumbling through a book looking up a subject when, by means of an index, it can be found at one turn. The work of the publishers is done with commendable taste and neatness.

